# Flavonoids Modulate the Accumulation of Toxins From *Aspergillus flavus* in Maize Kernels

**DOI:** 10.3389/fpls.2021.761446

**Published:** 2021-11-26

**Authors:** Lina Castano-Duque, Matthew K. Gilbert, Brian M. Mack, Matthew D. Lebar, Carol H. Carter-Wientjes, Christine M. Sickler, Jeffrey W. Cary, Kanniah Rajasekaran

**Affiliations:** United States Department of Agriculture, Agricultural Research Service, New Orleans, LA, United States

**Keywords:** *Aspergillus*, aflatoxin, GWAS, systems biology, *in planta*, flavonoids

## Abstract

*Aspergillus flavus* is an opportunistic fungal pathogen capable of producing aflatoxins, potent carcinogenic toxins that accumulate in maize kernels after infection. To better understand the molecular mechanisms of maize resistance to *A. flavus* growth and aflatoxin accumulation, we performed a high-throughput transcriptomic study *in situ* using maize kernels infected with *A. flavus* strain 3357. Three maize lines were evaluated: aflatoxin-contamination resistant line TZAR102, semi-resistant MI82, and susceptible line Va35. A modified genotype-environment association method (GEA) used to detect loci under selection *via* redundancy analysis (RDA) was used with the transcriptomic data to detect genes significantly influenced by maize line, fungal treatment, and duration of infection. Gene ontology enrichment analysis of genes highly expressed in infected kernels identified molecular pathways associated with defense responses to fungi and other microbes such as production of pathogenesis-related (PR) proteins and lipid bilayer formation. To further identify novel genes of interest, we incorporated genomic and phenotypic field data from a genome wide association analysis with gene expression data, allowing us to detect significantly expressed quantitative trait loci (eQTL). These results identified significant association between flavonoid biosynthetic pathway genes and infection by *A. flavus*. *In planta* fungal infections showed that the resistant line, TZAR102, has a higher fold increase of the metabolites naringenin and luteolin than the susceptible line, Va35, when comparing untreated and fungal infected plants. These results suggest flavonoids contribute to plant resistance mechanisms against aflatoxin contamination through modulation of toxin accumulation in maize kernels.

## Introduction

*Aspergillus flavus* is a saprophytic and opportunistic fungus that can infect multiple crops of economic significance such as cotton, maize, tree nuts, and peanuts. During seed development, *A. flavus* infection can lead to fungal production of several toxic secondary metabolites, including the polyketide-derived aflatoxins (Moellenbeck et al., 2001; Wu et al., 2014), as well as cyclopiazonic acid and aflatrem (Frisvad et al., 2019). In the United States, financial losses due to aflatoxin (AF) contamination have been estimated to be between $163 million and $500 million annually for maize, peanuts, and other crops (Wu, 2006; Mitchell et al., 2016). In developing sub-Saharan Africa, where regulatory controls are often ineffective, consumption of AF-contaminated foods is directly linked to liver disease, tumor development, stunted development in children, immunosuppression, and other abnormalities (reviewed in Wu et al., 2014).

The AF compounds produced by *A. flavus* include structurally similar chemical forms named B_1_, and B_2_ (Rigó et al., 2002; Kumeda et al., 2003). Several approaches have been employed to mitigate the impacts of aflatoxin contamination in maize. These include classic breeding techniques to increase fungal resistance (Brown et al., 2013), the development of genetically modified (GM) maize crops (Cary et al., 2011; Arias et al., 2015; Rajasekaran et al., 2018), and preharvest bioremediation (Senghor et al., 2019) by applying non-toxin-producing strains of *A. flavus* to crop fields, resulting in lower total AF accumulation (Ehrlich, 2014). The latter approach has resulted in several biocontrol products currently being used in several countries for controlling AF in maize, groundnuts, and cotton (Senghor et al., 2019).

Elucidating the molecular processes of resistance to aflatoxin contamination in maize remains important to developing mitigation strategies. Several high-throughput sequencing analyses have been conducted to determine the genes and potential proteins that influence crop host resistance in maize, particularly when colonized with *A. flavus* (Shu et al., 2015; Liu et al., 2021). Microarray analysis performed in two resistant and two susceptible lines showed differentially expressed genes located in previously identified quantitative trait loci (QTL) regions (Kelley et al., 2012). Other studies have identified genes correlated with *A. flavus* infection, such as 5,061 fungal genes (Dolezal et al., 2013) and 4,000 maize genes (Dolezal et al., 2014), which are differentially expressed after 96 h of infection. Among the differentially expressed genes in maize, several are involved in plant defense, signaling pathways, and potential disruption in kernel development (Dolezal et al., 2014). A quantative gene expression study that examined 94 genes that were previously linked to host resistance to *A. flavus* identified two major groups of maize lines: Group 1 showed high aflatoxin accumulation and low levels of gene expression, and Group 2 showed low aflatoxin accumulation and high levels of gene expression (Jiang et al., 2013). Results of these studies indicate that there are genes whose expression is correlated with resistant [R] and susceptible [S] maize lines (Luo et al., 2011; Kelley et al., 2012; Dolezal et al., 2013, 2014). Nevertheless, a comprehensive study that links transcriptomics and genomics data followed by functional analyses has yet to be undertaken to determine the biological role of these genes and proteins of interest in host resistance against this fungus.

Multiple studies have focused on understanding the natural genetic variation that contributes to maize resistance to aflatoxin accumulation, including studies that have detected QTLs in several chromosomal bins with hundreds of candidate genes (Warburton et al., 2011, 2015; Farfan et al., 2015). Association mapping using 300 inbred maize lines revealed a considerable amount of genetic and phenotypic variation for maturity, aflatoxin contamination, and other traits (Warburton et al., 2013, 2015). Another study that aimed to identify genomic regions associated with yield, resistance to aflatoxin contamination, and other important agronomic traits used 346 maize inbred lines to determine that the aflatoxin mitigation trait involved multiple loci (Farfan et al., 2015).

Systems genetics or genome-wide association studies (GWAS) can incorporate -omics information such as the expression or accumulation of transcripts, proteins, metabolites, and phenotypes to identify genes or other mechanisms associated with host resistance (Civelek and Lusis, 2014; Feltus, 2014). Furthermore, this analysis can be combined with known pathway and network data or developed into novel network identification (Wang et al., 2010; Califano et al., 2012; Ramanan et al., 2012; Dembeck et al., 2015). Such explicit pathway approaches using high-throughput sequencing data with GWAS may detect the enrichment of genes in a network even if individual associations do not attain genome-wide significance thresholds. This then results in refinement in the identification of candidate loci during fine mapping (Pickrell, 2014; Rodgers-Melnick et al., 2015; Kremling et al., 2019). Using bioinformatic approaches such as these, systems genetics can greatly improve the ability to find and understand the genes and pathways responsible for complex trait variation in plants.

Here, we report a system and pathway GWAS bioinformatics data analysis approach using RNA sequencing data of aflatoxin contamination resistant (TZAR102), semi-resistant (MI82), and susceptible (Va35) maize lines inoculated with *A. flavus* and then combined with previously published GWAS data on aflatoxin contamination (Warburton et al., 2015). Our bioinformatics analysis identified several candidate loci and pathways of interest associated with the molecular mechanisms of maize resistance to aflatoxin accumulation, such as cellular transport, vesicular and lipid droplet formation, production of flavonoids, including flavones and anthocyanins, and differential accumulation of pathogenesis-related (PR) genes and tetraspanins. In this study, we report *in planta* functional evidence that flavonoid accumulation in resistant maize lines correlates well with lower accumulation of aflatoxin.

## Materials and Methods

### Fungal Strains and Maize Lines

*Aspergillus flavus* strain NRRL 3357 is described by Nierman et al. (2015), herein called *A. flavus*, and was grown at 31°C on V8 medium [5% V8 Vegetable Juice (Campbell Soup Company, Camden, NJ, United States), 2% agar, pH 5.2]. To prepare inoculum, conidia from 6-day-old cultures were suspended in 0.02% Triton X-100; the conidial concentration was determined with a hemocytometer and adjusted to 4 × 10^6^ conidia ml^–1^. Maize lines used include the aflatoxin contamination susceptible line Va35 (USDA NPGS Acc. PI587150), resistant line TZAR102 (USDA NPGS Acc. PI 654049) (Brown et al., 2013; Brown and Goldman, 2016), and semi-resistant line MI82 (Brown and Goldman, 2016).

### Maize Kernel Infection Assay

Maize kernels from the three maize lines described above were inoculated with *A. flavus* NRRL 3357. This assay is based on the kernel screening assay (KSA) as previously described (Brown et al., 1993). Briefly, dehydrated kernels were sterilized with 70% ethanol and soaked in an inoculum of 4 × 10^6^ fresh spores/ml in distilled water of *A. flavus* NRRL 3357 for 3 min. The same procedure was applied to the mock-treated kernels, but no *A. flavus* spores were added to the distilled water. Kernels were removed from inoculum and incubated at 31°C in the dark. *A. flavus*-infected maize kernels were collected at 8 h, 3 days, and 7 days post inoculation. The kernel exterior was cleaned with deionized water to remove external mycelia. Kernels were then frozen in liquid nitrogen and stored at −80°C.

### RNA Extraction for High-Throughput Sequencing

Kernel samples were ground with 0.5-mm diameter zirconia-silica beads (BioSpec Products, Bartlesville, OK, United States) using a TissueLyser II (Qiagen, Germantown, MD, United States) for RNA extraction. Total RNA was extracted from ground samples using QIAzol Lysis Reagent (Qiagen), following the miRNeasy Mini Kit manufacturer’s protocol (Qiagen). For DNase treatment, RNA was treated with PureLink DNase (ThermoFisher, Waltham, MA, United States). After the extraction, quality and quantity of RNA were confirmed using a 2100 Bioanalyzer with Agilent RNA 6000 Nano Kit (Agilent, La Jolla, CA, United States). A total of 1 μg of RNA was used to do further DNase treatment using TURBO DNase (Thermofisher). mRNA was then isolated with Dynabeads Oligo (dT) 25, using three rounds of isolation per the manufacturer’s protocol. RNA libraries were prepared with 10- to 50-ng purified mRNA using NEBNext Ultra directional RNA library Prep Kit for Illumina (New England BioLabs, Ipswich, MA, United States), following the manufacturer’s protocol. The library size was approximately 450 bp as measured using 2100 Bioanalyzer with an Agilent High Sensitivity DNA kit (Agilent). Concentrations of the samples were measured using a Qubit dsDNA HS kit (ThermoFisher) on a Qubit fluorometer. A total of 48 libraries were combined into two pooled samples at a final concentration of 1.8 pM each and sequenced using an Illumina NextSeq 500 sequencer in a high-output mode, providing 909 million paired-end (PE) reads (2 × 150 bp). The raw reads were submitted to NCBI and can be accessed under BioProject PRJNA767817.

### High-Throughput Sequencing Data Analysis

RNA extractions contained material from maize and fungi, so the sequencing reads were competitively aligned with the concatenated *Zeamays* B73 genome (version 4.35 from Gramene) and *A. flavus* NRRL 3357 genome (JCVI-afl1-v2.0; GCA_000006275.2). STAR (version 2.7.3a) was used to align the reads with the following settings:–alignIntronMax 60000–outFilterMismatchNoverReadLmax 0.15–outFilterMismatchNmax 23–outFilterMultimapNmax 20–twopassMode Basic. Reads mapping to genes were counted using feature Counts (version 1.5.2) (Liao et al., 2014) with the settings: “-a -p -s 2 –primary.”

Raw counts from each sample were processed using DESeq2 (v1.28.1); briefly, the counts were normalized by the dispersion estimates and library size factors (Anders and Huber, 2010). To determine gene expression differences, we used an additive model that included the following factors: genotype (Va35: V, TZAR102: T, and MI82: M), treatment (Treated: Fungus: F and untreated: mock: m) and time (8 h, 3 and 7 days). Statistical analyses for each individual factor were performed using DESeq2 (v1.28.1) with a Wald test and false discovery rate adjustment for the *p*-values (Anders and Huber, 2010). For the contrasting comparisons in genes expression, we used the following: genotype, Va35 vs. TZAR102, Va35 vs. MI82; treatment, untreated vs. treated; time, 8 h vs. 3 days and 8 h vs. 7 days. To examine the effect of fungal treatment within the different maize lines in more granular detail, an additional model was used with DESeq2 using a single term, which was a concatenation of the three terms above. Pairwise comparisons between treated and untreated samples were then made for each maize line at each time point. Genes were considered differentially expressed if the absolute value of log2 fold change was greater than 1 and the adjusted *p*-value was less than 0.05. Normalized counts were used for further analysis, and all the boxplots were generated using R and the log base 2 of count values.

### Metabolic Pathway Visualization

Normalized count data for each biological replicate were averaged and used for generating metabolic pathway views by using pathway tools software V23.0 and an omics dashboard tool (Karp et al., 2002, 2015; Latendresse and Karp, 2011). We generated graphs from the omics dashboard from the online tool by using a logarithmic scale for the expression data and the sum of all data values in each metabolic pathway category. In each graph, the displayed expression abundance was the cumulative effect of many small changes thus showing the way the cell is switching toward a specific metabolic activity in each sample.

### Multivariate Linear Analysis

To explore the general variation of gene expression data, a redundancy analysis (RDA) was performed to determine multivariate linear associations between the normalized gene expression counts and the variables influencing their expression: maize line (Genotype: Va35, MI82, and TZAR102), fungal treatment (treatment: untreated and treated), and time after inoculation (time: 8 h, 3 days, and 7 days) by using R V3.6.1 (R Core Team, 2015). The RDA analysis is a modified version of a genotype-environment association method (GEA) used to detect loci under selection (Forester et al., 2018). The modified RDA method from Forester et al., 2018 was done by replacing the genotype variable with the gene expression matrix and replacing its various environments with maize line, fungal treatment, and time after inoculation matrixes. This type of modified GEA-RDA analysis has been performed in rice by using gene expression data and independent variables such as flooded and non-flooded environments and different rice genotypes (Castano-Duque et al., 2021). These modifications allowed us to detect the groups of expressed genes that are significantly influenced by the maize line, treatment, and time simultaneously. An ANOVA was performed on the RDA model to determine the significance of each independent factor in the model by using 999 permutations to reduce the false discovery rate. Finally, to determine the genes that are highly influenced by the multivariate explanatory variables (maize line, fungal treatment, and time after inoculation) in the model, the loadings of the genes in the RDA ordination space were scanned using two significant RDA axes to determine which genes are within three standard deviations (two tailed *p*-value = 0.0027) within the genes loading values distribution. The genes that are closer to the center of the distribution tend to have low or no correlation with the maize line, treatment or time, while those in the tails of the distribution are most likely significantly influenced by the independent variables of the experiment (Legendre and Legendre, 1998; Forester et al., 2018). To detect directionality of the correlation of the expression of the genes in relation to the independent variables, further analyses were performed using DESeq2. Also, to determine biological enrichment of these significant genes, a gene ontology enrichment analysis was performed.

### Gene Ontology Enrichment

The genes that passed the standard deviation cut-off value from the RDA were analyzed using a singular ontology enrichment analysis AgriGO V2.0 online annotation tool (Du et al., 2010; Tian et al., 2017). In short, we provided a list of genes/probes names (genes that passed standard deviation cut-off), and enriched GO terms were computed using Fisher’s Exact Test using the maize genome locus (maizesequence.org, with 25,288 genes annotated) as background followed by the Yekutieli (FDR) multi-test adjustment method for *p*-values. Functional enrichment analysis was done to test for enrichment of GO terms and CornCyc pathways within differentially expressed genes and co-expression modules using GOseq (Young et al., 2010). The *p*-values were corrected for multiple testing using the Benjamini and Hochberg method, and the adjusted *p*-values were considered enriched if they were less than 0.05.

### Co-expression Analysis

Co-expression networks were individually created for each of the three maize lines using the variance stabilized mRNA counts from DESeq2 as input for WGNCA (Langfelder and Horvath, 2008). The network adjacency matrix was created with the settings “corFnc = ‘bicor’, type = ‘signed hybrid’, power = 12.” Module preservation analysis was done using the module Preservation function, comparing the MI82 and Va35 modules with the TZAR102 modules as the reference. Heatmaps were made using the tidy Heatmap R package (Mangiola and Papenfuss, 2020) with the regularized log transformed counts from DESeq2.

### Linkage of Genome-Wide Association Analysis With High-Throughput Sequencing Data

We performed a genome-wide association analysis (GWAS) on published phenotypic data for aflatoxin concentrations measured on 300 maize genotypes (Warburton et al., 2015). The phenotypic data used were the logarithm of LS means of aflatoxin concentrations calculated as described and provided by Warburton et al. (2015). We further performed a transformation step on the LS means to achieve data normality (Star09LSM^2, Star10LSM^3, CSta09LSM, CSta10LSM, Lubb09LSM^3, and Lubb10LSM^2). We ran GWAS on GAPIT V3.0 (Lipka et al., 2012) by using 405,648 the single nucleotide polymorphisms (SNP) data base [provided by Warburton et al. (2015) and filtered to keep the major and minor alleles asbinary type], a mixed linear model, a kinship matrix (generated by GAPIT using the vanRaden method), and six principal components (generated by GAPIT).

### Single Nucleotide Polymorphisms-to-Gene and Gene-Set Analysis

We performed a gene-set analysis using MAGMA V1.05 (de Leeuw et al., 2015) by assigning SNPs to genes, taking into account a linkage disequilibrium of 2.5Kb windows at each flanking side of the annotated maize reference genome (V4, gramene.org accessed January 2020). This analysis used 404,860 SNPs out of the 405,648 SNPs (provided by Warburton et al., 2015), and 28,297 annotated genes containing valid SNPs in genotype data. We performed an association analysis of the SNP-to-gene data and the GWAS *p*-values, followed by a *p*-value estimation based on 1,000 permutations to correct for multiple testing errors. Finally, we used MAGMA to generate a meta-analysis that included all sites and seasons by using the weighted Stouffer’s Z method (Stouffer et al., 1949) to combine *p*-values from independent statistical tests.

### Dense Module Search for Genome-Wide Association Studies

We linked the gene identities of the meta-analysis results from MAGMA to a pre-built protein-protein interaction (PPI) network from maize (Musungu et al., 2015). We added to the network analysis the gene expression data as a *case-control* experiment where the *case* was TZAR102 (fungus treated) and *control* was Virginia35 (Va35) (fungus treated) and performed a dense module search (dmGWAS R package) (Wang et al., 2015) in R V3. 9.1. The dense module algorithm generated a 100-protein subnetwork based on *p*-values from MAGMA and the gene expression values. Networks were visualized using Cytoscape V3.7.2, and the fold change ratios of gene expression with their corrected *p*-values were used to determine highly activated areas of the network (Ideker et al., 2002). Finally, to determine proteins of interest within the highly activated areas of the network, we used the gene expression plasticity to determine which genes were stress response linked (high plasticity) and which were housekeeping linked (low plasticity) (Xiao et al., 2019).

### *In planta* Experiments

Maize plants were grown in a BSL-2 greenhouse at the USDA-ARS Southern Regional Research Center in New Orleans, Louisiana. The greenhouse bays were equipped with supplemental lights to provide 14-h/8-h day/night cycle at 80°/70°C. *In planta* fungal inoculations were performed ∼20 days after pollination when the kernels were in the milk stage. Infection with *A. flavus* spore suspension (1 × 10^6^ conidia ml^–1^) was done by puncturing the kernels to a depth of ∼5 mm with a sewing needle dipped in the spore suspension (Supplementary Figure 3), otherwise known as the pin-prick technique, which has been established as the most efficient inoculation technique *in planta* (maize plants) and *in field* conditions (Zummo and Scott, 1989). Kernels were collected 3 days post-inoculation, flash frozen, and stored at −80°C for further analysis.

### RNA Extraction for Quantitative Real-Time PCR Analysis

We evaluated the pattern of gene expression from the genes of interest by using whole kernels collected from the *in planta* experiments. Whole kernel samples (*N* = 3) were ground using a ball-mill tissue Geno/Grinder (SPEX SamplePrep, Metuchen, NJ, United States) two times for 30 s at 2,000 strokes/min under liquid nitrogen. RNA was extracted using QIAzol Lysis Reagent (Qiagen), following the miRNeasy Mini Kit manufacturer’s protocol (Qiagen). RNA content was measured using a Nanodrop (ThermoFisher Scientific), and cDNA was made using High-Capacity cDNA Reverse Transcription Kit (ABI, Foster City, CA, United States), following the manufacturer’s instructions. Quantitative real-time PCR (qRT-PCR) analyses were done using Tubulin (F ACA CCA TTG GGA GTC TA; R TTG TGG GGA CCA CTA CTT TC) primers for the endogenous control. We tested the gene expression of flavone synthase (Zm00001d029744_T001; F CTC TTC AGA ACC TAG CGA ATC G; R ATG GAC AAA CAT TGC AGA ACG). The PCR conditions used were 95°C for 3 min, and then 40 cycles of 95°C for 10 s, 55°C for 10 s, 72°C for 30 s, followed by cooling (Bio-Rad CFX96 Real-Time system). The relative quantification values were obtained by using Bio-Rad CFX manager (version 3.1). Data were analyzed with the R V3.9.0 (Agricolae and dplyr packages) (R Core Team, 2015) by using logarithmic normalization transformations, and then performing a multiple-factor ANOVA, followed Tukey pairwise comparison post-test to discriminate treatment means by honest significant difference (HSD).

### Flavonoid Extractions

Harvested samples were grounded with 0.5-mm diameter zirconia-silica beads (BioSpec Products, Bartlesville, OK, United States), using a TissueLyser II (Qiagen, Germantown MD) for RNA extraction. Ground tissue was lyophilized (VirTisFreezemobile 25EL Freeze Drying System) overnight, and then metabolites were extracted overnight with shaking by using a 0.01 mg/ml TBHQ in methanol solution. The mixture was centrifuged at 15,000 rpm for 5 min, and the supernatant was transferred to a new tube and kept at –20°C for further analysis.

### Aflatoxin Analysis

Each extract (from ∼200-mg ground, lyophilized maize seed) was redissolved in methanol (1.5 ml) and centrifuged to remove particulate. The supernatant was analyzed using a Waters ACQUITY UPLC system (40% methanol in water, BEH C18 1.7 μm, 2.1 mm × 50 mm column) using fluorescence detection (Ex = 365 nm, Em = 440 nm). Samples were diluted 10-fold if the aflatoxin signal saturated the detector. Analytical standards (Sigma-Aldrich, St. Louis, MO, United States) were used to identify and quantify aflatoxins: aflatoxin B1 (AFB_1_) and aflatoxin B2 (AFB_2_). Aflatoxin content was expressed in ng/g (ppb) fresh weight of homogenized kernels.

### Flavonoid Analysis

Each extract (from ∼200-mg ground, lyophilized maize) was redissolved in methanol (0.5 ml) and centrifuged to remove particulate matter. Samples were analyzed on a Waters ACQUITY UPLC system, coupled to a PDA UV detector and a Waters Xevo G2 XS QTOF mass spectrometer controlled by a MassLynx workstation using the following conditions: Waters BEH C18 1.7 μm, a 2.1 mm × 50 mm column, 0.5 ml/min, 1-μl injection volume, solvent A (0.1% formic acid in water); solvent B (0.1% formic acid in acetonitrile); 5% B (0 –1.25 min), gradient to 75% B (1.25–4.75 min), gradient to 100% B (4.75–5.0 min), 100% B (5.0–7.5 min), and then column equilibration 5% B (7.6–10.1 min). MS^*E*^ continuum data (50–800 m/z) were collected in a negative ion mode using collision energy alternating between low (7 eV) and high energy (linear ramp from 15 to 40 eV). Peaks were identified using authentic standards (naringenin, luteolin, luteolin 7-glucoside, all purchased from Sigma-Aldrich) and quantified using Waters UNIFI software.

## Results

### Gene Expression Analysis From Kernel Screening Assays Reveals Putative Maize Defense Pathways in Response to Fungal Infection

**FIGURE 1:**
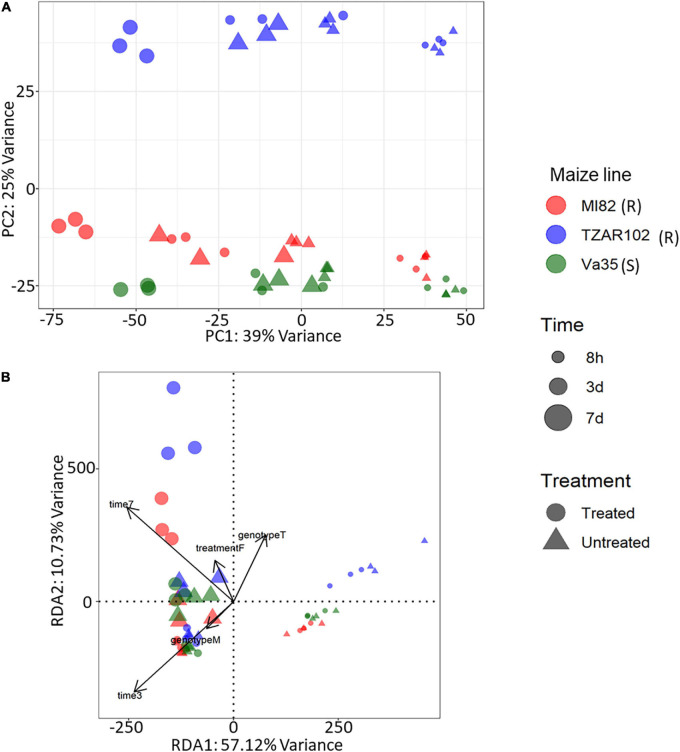
Gene expression profiles from KSA using **(A)** principal component analysis, x-axis explained 39% of variance and y-axis explained 25% of variance. PCA was performed using rlog-transformed count data from DESeq2. **(B)** Expression profiles using redundancy analysis model, x-axis explained 57.1% of variance and y-axis 10.73% of variance. RDA was performed using rlog-transformed count data from DESeq2. PCA and RDA were performed using three corn lines and three time points of treated (Fungus) and untreated (Buffer) samples. Corn lines are identified by color, red, MI82 (R – Resistant); blue, TZAR102 (R – Resistant); green, Va35 (S – Susceptible). Figure shape represents treatment, circle, treated (Fungus) and triangle, untreated (Buffer). Time post treatment is represented in the size of the shapes, smallest, 8h; medium, 3d; largest, 7d.

Our differential expression analysis showed that time after fungal treatment had the greatest effect on gene expression in terms of the total number of differentially expressed genes (DEGs) with 5,860 DEGs from the 7 vs. 3 days comparison and 10,804 DEGs from the 3 days vs. 8 h comparison. Fewer differences in gene expression were observed between maize lines (Supplementary Table 1). Comparison of treated (inoculated with *A. flavus*) vs. untreated (mock infected with buffer) samples showed a total of 3,680 genes with significantly different expression (Adjusted *p*-value < 0.05 and absolute log2 fold change > 1), of these 3,680 genes, 1,515 were upregulated in fungal inoculated samples (Supplementary Table 1); further gene ontology analysis was performed to understand the types of genes with higher expression in response to fungal treatment. To understand the overall variation in our data, we performed a principal component analysis (PCA) (Figure 1A) of the rlog counts from each sample. The first principal component, which explained 39% of the variance, showed a clear grouping of the samples according to a time point. The second principal component explained 25% of the variance and had a grouping of the samples according to maize line with Va35 [S] and MI82 [R] clustered closer than TZAR102 [R].

We performed a pathway overview of maize metabolism during fungal infection by integrating and comparing the gene expression of TZAR102 and Va35. We were able to detect an overall activation post-inoculation of the L-ornithine biosynthetic pathway (Supplementary Figure 1) in TZAR102. One of the genes involved in this pathway, acetylornithine deacetylase (Zm00001d01767), showed significantly increased gene expression on TZAR102 at 7 days post infection compared with Va35 and MI82 (Supplementary Figure 1). Acetylornithine deacetylase is an enzyme that catalyzes the production of L-ornithine from *N*-acetyl-L-ornithine, a preliminary step to arginine biosynthesis. Arginine has the highest N:C ratio among all amino acids and is a building block for polyamines, such as putrescine, spermidine, and spermine (Srivastava et al., 2007; Neily et al., 2011). These polyamine compounds have been shown to play a vital role in plant resistance to fungal growth (Majumdar et al., 2019). The late activation (7 days) of polyamine precursors that we observed could suggest a secondary, long-term defense response in the plant.

Gene expression patterns can be affected by a combination of variables. We examined the multivariate relationships of infection (treated compared with untreated), time (3, 5, 7 days), and maize line (susceptible, resistant) on gene expression by performing a redundancy analysis (RDA). RDA allowed us to associate the gene expression with explanatory variables like genotype, treatment, and time by performing multivariate regression modeling (Legendre and Legendre, 1998). We determined that all the explanatory variables significantly covary with gene expression, meaning that differences in maize line, treatment, and time can significantly influence the overall gene expression patterns (Table 1). Using the first two components of the RDA (Percentage of variance explained by RDA1 = 57.12 and RDA2 = 10.73), we illustrated the multivariate linear relationships among maize line, treatment, and time by measuring the angles between explanatory variables vectors (Figure 1B). The strength of the relationship between variables is determined by the cosine of the right-handed projected angle between vectors (Explanatory variables and/or response variables) (Legendre and Legendre, 1998). Our results showed that fungal treatment is positively correlated with TZAR 102 line and 7 days post infection (Angle is < 90°; cos < 90° = positive value), meaning that high positive gene expression changes tend to be correlated with fungal treatment in TZAR 102 and 7 days.

**TABLE 1:** Redundancy analysis results using a linear covariate model and analysis of variance.

Variable	df*	Variance	F*	Pr (>F)*
Genotype	2	3.95E + 09	9.4673	0.001***
Treatment	1	1.35E + 09	6.4887	0.003**
Time	2	2.64E + 10	63.1942	0.001***
Residual	48	1.00E + 10		

*df, degrees of freedom; F, Fisher’s statistic value; Pr(>F), *P*-value associated with the F statistic. **Significant (P ≤ 0.05), ***Significant (P ≤ 0.001).

To determine the genes that show significant changes in expression due to covariation of maize line, treatment, and time variables, we used the RDA loadings distributions from the first two RDA axes. The genes selected have RDA loading values above three standard deviations from the expression mean (two tailed *p* = 0.0027) (Supplementary Table 2). Using this *p*-value cut-off limit, we selected 295 genes and performed a GO enrichment analysis that showed enrichment of pathways linked to response to fungi and microbes (Supplementary Figure 1). The “defense response to fungus” GO category included genes such as, hevein-like preproproteins that tend to be fungal growth inhibitors known as pathogenesis-related (PR) proteins (Zm00001d048947, Zm00001d048948, Zm00001d048949, and Zm00001d048950, Supplementary Table 3). The analysis revealed another significant GO category, “lipid particle,” that included genes linked to generation of lipid bilayers (AC206941.2_FG002, GRMZM2G333069, GRMZM2G480954, GRMZM2G410152, Supplementary Table 2). Genes involved in lipid bilayer generation could play a role in transport of proteins or metabolites that could be involved in defense responses against fungi (An et al., 2007; Colombo et al., 2014).

The differentially expressed genes for maize-treated-vs.-untreated comparisons were enriched for the CornCyc pathways of eriodictyol C-glucosylation, naringenin C-glucosylation, apigeninidin 5-*O*-glucoside biosynthesis, and luteolinidin 5-*O*-glucoside biosynthesis, and initial reactions in the phenylpropanoid biosynthesis pathway (Supplementary Table 4). Examining pairwise comparisons between treated and untreated maize lines at each time point showed that the GO term “defense response to fungus” was highly enriched for all maize lines at days 3 and 7, but, for the 8-h comparisons, was only enriched for the two resistant lines MI82 and TZAR102. This could indicate that the early activation of genes in this category upon infection could contribute to the resistance trait. Likewise, the GO terms for “detection of biotic stimulus” and “regulation of response to biotic stimulus” were enriched in most comparisons but, for the 8-h comparison, were only enriched in TZAR102. The GO term “flavonol biosynthetic process” was highly enriched for MI82 and TZAR102, but not for Va35. The GO term “flavone synthase activity” was enriched for all corn lines at the 7-day time point but was only enriched for MI82 and TZAR102 at the 3-day time point. Co-expression analysis was conducted on the variance-stabilized mRNA counts using WGCNA (Langfelder and Horvath, 2008). Co-expression networks were individually created for each of the three maize lines, and the modules identified were then examined for how well they were preserved in the other lines. Two modules identified in TZAR102 that were positively correlated with fungal treatment were highly preserved in MI82 but not preserved in Va35. The two modules, light yellow and maroon, consist of 252 and 321 genes, respectively. The Z-score summary preservation statistic from WGCNA for the light yellow module was 18.8 for MI82 and only 7.0 for Va35. For the maroon module, the preservation Z-score was 13.6 for MI82 and 2.2 for Va35 (Supplementary Table 4). AZ-summary greater than 10 is considered strong evidence for preservation, and a score between 10 and 2 suggests moderate-to-weak evidence for preservation (Langfelder et al., 2011).

Among the genes within the light yellow module is flavone synthase type 1 (fnsi1; Zm00001d029744), which has a high module membership value of 0.92 and is also found within the dmGWAS network. The maroon module also contained two additional genes within the flavonoid biosynthesis pathway, Zm00001d001849 and Zm00001d027534, both annotated as having 4-coumarate-CoA ligase enzymatic activity in CornCyc and having module membership values of 0.53 and 0.80, respectively. Functional enrichment analysis showed that the light yellow module is enriched in the genes in the Reactome pathway for jasmonic acid signaling, the CornCyc pathway for apigeninidin 5-*O*-glucoside biosynthesis, and the GO term for “response to fungus.” The maroon module was enriched for the GO term for “respiratory burst involved in defense response” (Supplementary Table 4).

### Linkage of Genome-Wide Association Analysis of Aflatoxin Accumulation and Gene Expression Data Reveals a Maize Defense Mechanism That Links Cellular Transport and Production of Flavonoids

We analyzed previously published field phenotypic data of aflatoxin accumulation levels from 300 maize lines cultivated in 2009 and 2010 in three different locations of southern US (Warburton et al., 2015). To these data, we incorporated gene expression data from our KSA using TZAR102 and Va35 maize lines to determine metabolic pathways significantly correlated with the aflatoxin accumulation trait. We used only the gene expression from TZAR102 and Va35 because one has a resistant background while the other one has the susceptible background, thus using a *case-control* experimental design for post-GWAS analysis can be applied (Case for resistant genotype and control for susceptible genotype). Our GWAS showed similar results when compared with the published GWAS by Warburton et al. (2015) (Supplementary Figure 2). The Manhattan plots showed several SNPs with low effect throughout the genome, which is indicative of a polygenic trait (Daub et al., 2013; Corwin and Kliebenstein, 2017). There was variation in SNP association values between years 2009 and 2010, which could be due to differences in environmental factors that were addressed by Warburton et al. (2015) and shows the high plasticity of the trait (van der Sijde et al., 2014). To determine which genes were associated with resistance to aflatoxin accumulation, we used the GWAS results to perform a generalized gene-set analysis using Multi-marker Analysis of GenoMic Annotation (MAGMA, Supplementary Figure 2) (de Leeuw et al., 2015). MAGMA takes into consideration linkage disequilibrium (LD) by linking the SNPs in 2.5-Kb windows to the corresponding genes in those regions from the *Z. mays* reference genome (de Leeuw et al., 2015). MAGMA results showed similar Manhattan plots to those obtained from GWAS (Supplementary Figure 2); thus, these results reinforced that the trait is polygenic and there is a high degree of variation between sites and year.

**FIGURE 2:**
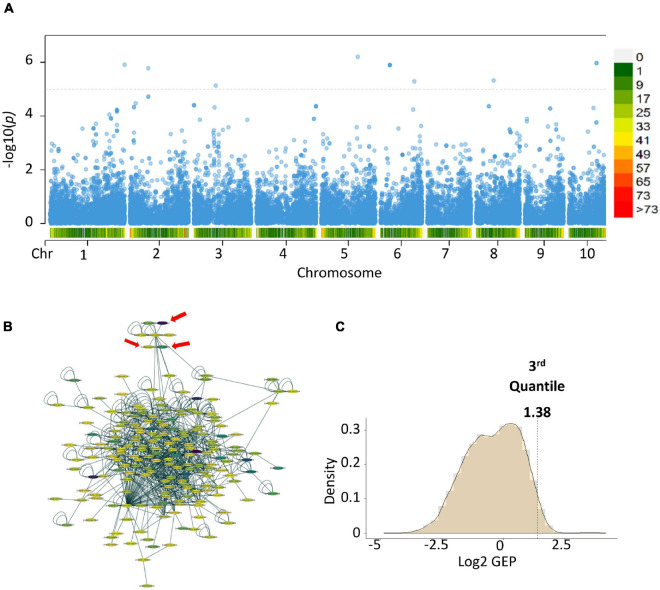
Post-GWAS results of aflatoxin accumulation generated using Warburton et al. (2015) data and KSA gene expression data. **(A)** Meta-analysis of the MAGMA results from the College Station, Lubbock and Starkville results. **(B)** Subnetworks with the highest score from the top 100 modules created by using dense module network search (EW_dmGWAS) in R. **(C)** Density plot of the gene expression plasticity data from the rlog counts of the RNA-seq data. In the Manhattan plot, the horizontal dotted lines are the thresholds for significant log 10 *p*-value/Number of markers), below chromosome labels on the *x*-axis is the gene density in the chromosomal location. In the network, color represents the gene expression plasticity values.

To link the gene-set MAGMA results from the different locations and seasons, we performed a meta-analysis (de Leeuw et al., 2015). Meta-analysis showed 12 genes with a *p*-value ≤ 7.28 × 10-06 (Figure 2) (Zm00001d016150, Zm00001d025276, Zm00001d025277-Serine carboxypeptidase-like51, Zm00001d034707, Zm00001d035642-Glutathione S-transferase T1, Zm00001d035643, Zm00001d035644-ATP- dependent DNA helicase, Zm00001d035645, Zm00001d003990-

Purple acid phosphatase 3, Zm00001d009564-Guanylate-binding family protein, Zm00001d037776, and Zm00001d040999). Gene-set analysis performed on the meta-analysis results showed two gene ontology terms highly associated with the trait of interest, Set 1: GO:0005794 Golgi apparatus (No. of genes = 503, *p*-value = 0.000496223) and Set 2: GO:0016020 membrane (No. of genes = 5,174, *p*-value = 0.00034836). Genes and proteins linked to the Golgi apparatus tend to be involved in vesicle trafficking or cellular communication (Skotland et al., 2017).

**FIGURE 3:**
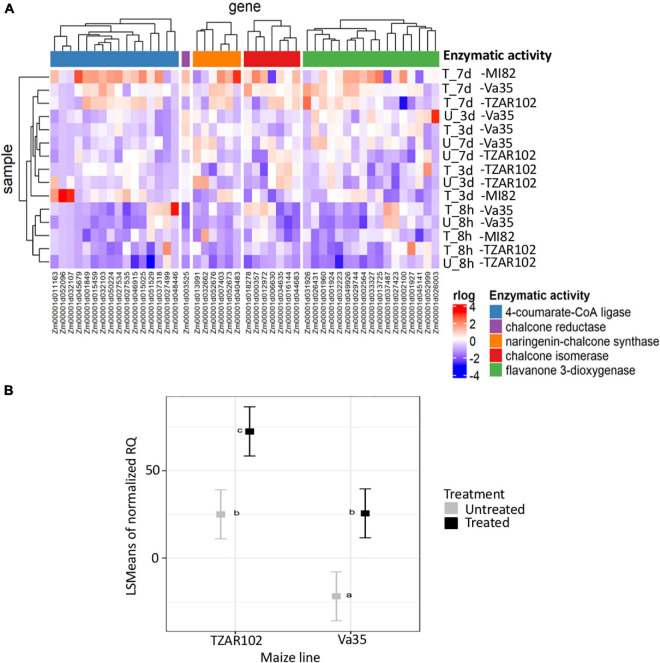
Flavonoid pathway analysis. **(A)** Hierarchical clustering of gene expression patterns for flavonoids biosynthesis metabolic pathways detected in TZAR102 (R-Resistant), MI82 (R-Resistant) and Va35 (S-Susceptible). **(B)** Square root of the least-square means of gene expression of flavone synthase from *in planta* experiment. The heatmap was built using rlog of counts generated with DEseq2, flavonoid pathway from CornCyc with genes grouped according to their enzymatic activity. The colors in the heatmap represent the scaled rlog values. T, Treated, U, Untreated. Metabolic pathways are labeled with different colors, blue is 4-coumarate-CoA ligase, purple is chalcone reductase, orange is naringenin-chalcone synthase, red is chalcone isomerase and green is flavanone 3-dioxygenase. For gene expression, TZAR102 and Va35 are compared in treated and untreated environments by two-way ANOVA. Letters above square plots indicate the HSD-Tukey test results with *P* < 0.05. Error bars represent standard errors of means.

We linked the gene identities of the meta-analysis results to a pre-built protein-protein interaction (PPI) network from maize (Musungu et al., 2015). We then added gene expression data using a *case-control* experimental set-up, where the *case* was TZAR102 (Fungus treated) and *control* was Va35 (Fungus treated) and performed a dense module search (dmGWAS R package) (Wang et al., 2015; Figure 2). The module dense algorithm generated a 100-protein subnetwork based on *p*-values from MAGMA and gene expression data. Proteins in these networks are capable of physically interacting with each other, showed high correlation with the trait of interest, and could have significant gene expression values. Finally, we detected significantly activated hubs (Ideker et al., 2002) within the 100-protein subnetwork using the RNA-seq data *p*-values when comparing treated with fungus against untreated (Figure 2). The most significantly activated hub had 188 protein nodes with 739 connections. We used the gene expression plasticity values (Figure 2) to determine which genes were stress response linked (high plasticity) and which were housekeeping linked (low plasticity) (Xiao et al., 2019). Some of the genes of interest with high plasticity values were genes encoding for enzymes involved in the flavonoid biosynthetic pathway (Zm00001d029744, Zm00001d044122, Zm00001d052492, and Zm00001d011438) (Figure 3). Flavonoids, such as flavonols, flavones, and flavanones, are phytochemicals with a benzo-γ-pyrone structure that play a wide variety of biological roles in plants and other organisms (Kumar and Pandey, 2013; Mierziak et al., 2014; Hostetler et al., 2017).

To determine the dynamics of flavonoid production in aflatoxin contamination-resistant and susceptible maize lines during *A. flavus* infection, we looked at the gene expression patterns of flavonoid metabolism genes (Figure 3 and Supplementary Table 5). We saw an increase in expression of genes with enzymatic activities, such as naringenin-chalcone synthase, and chalcone isomerase4-coumarate-CoA ligase, pointing at an increase in flavonoid production. This increase in expression was highest at 7-day post-fungal treatment for TZAR102, MI82, and Va35 (Figure 3). Despite the overall increase in expression in relation with time of the flavonoid pathway, there were genes with chalcone reductase and flavanone 3-dioxygenase enzymatic activity that showed lower expression in TZAR102 compared to Va35 after 7 days of infection (Figure 3). These opposing expression trends among genes in the flavonoid pathway could indicate that different metabolite intermediates in the pathway could accumulate to different levels in response to *A. flavus* infection.

**FIGURE 4:**
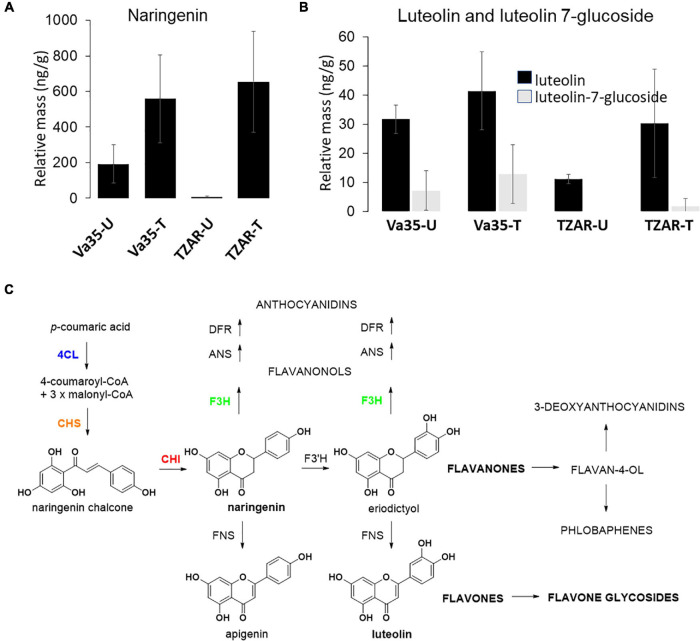
*In planta* flavonoids content of **(A)** naringenin, **(B)** luteolin and luteolin 7-glucoside levels in susceptible (Va35) and resistant (TZAR) maize lines (U, Untreated, T, Treated), **(C)** schematic of flavone biosynthesis.

Gene expression of flavone synthase (Zm00001d029744) from *in planta* experiments showed significantly higher levels in TZAR102 treated compared with Va35 treated with fungus (Figure 3). To determine if these gene expression patterns affected flavonoid metabolite production and mycotoxin accumulation *in planta*, we measured aflatoxin and flavonoid levels (Figure 4 and Supplementary Figure 3). We found that the resistant line (TZAR102) accumulates less aflatoxins (AFB_1_ and AFB_2_) than the susceptible line (Va35), and both lines showed an overall increase in flavonoid production concurrent with fungal infection (Figure 4 and Supplementary Figure 3). We observed that the naringenin fold ratio of control to infected is bigger in TZAR102 (100.88-fold ratio, F:C) than in Va35 (2.9-fold ratio, F:C) (Figure 3). Interestingly, luteolin and luteolin-7-glucoside accumulate in higher levels in Va35 untreated compared with untreated TZAR102, and their fold ratio of untreated vs. treated is not as high as naringenin (Luteolin: 1.3 Va35 and 2.5 TZAR102; Luteolin-7-glucoside: 1.6 Va35 and 1.8 TZAR102) (Figure 4). This could mean that naringenin, a precursor of glycosylated flavonoids and anthocyanins, could have an effect in modulating aflatoxin contamination in resistant maize lines.

## Discussion

Our study revealed activation of several important pathways and processes during *A. flavus* infection of maize, including arginine biosynthesis, extracellular vesicle production, and flavonoid biosynthesis. We observed gene activation leading to arginine biosynthesis in maize lines resistant to *A. flavus* infection (Supplementary Figure 1). Arginine has the highest N:C ratio among all amino acids, and it is a building block for polyamines, such as putrescine, spermidine, and spermine (Yousefi et al., 2019). These polyamine compounds might be playing a vital role in plant resistance to fungal growth (Majumdar et al., 2019). GO enrichment of RDA significant genes showed several PR genes that have been linked to plant defenses against fungal infection and other biotic stresses. GO categories included genes with a hevein-like preproprotein description that tend to be fungal growth inhibitors known as PR proteins (Supplementary Table 3) and the GO lipid particle category that included genes linked to generation of lipid bilayers (Supplementary Table 2). Genes involved in lipid bilayer generation could play a role in transport of proteins or metabolites involved in defense responses against fungi.

Lipid bilayer particles or extracellular vesicles (EVs) released from plant cells may play a role in communication, host-defense responses, and defenses against pathogen. For example, *Arabidopsis* plants infected with *P. syringae* produced high amounts of EVs, which carried proteins involved in abiotic and biotic stress responses (Rutter and Innes, 2017). Also, the lipid composition of the EVs might be involved in recognition events between two interacting organisms and/or viruses and potential downstream responses (Skotland et al., 2017). Our gene expression data from TZAR102, MI82, and Va35 linked with previously published phenomic and genomic association data (Warburton et al., 2015) showed enrichment in Golgi apparatus, and membrane-linked pathways show an association of vesicular trafficking with resistance to aflatoxin accumulation.

In addition to Golgi apparatus and membrane-linked pathways, our post-GWAS assessment showed that the flavonoid biosynthetic pathway is linked to maize responses to *A. flavus* infection. Flavonoids are phytochemicals with a benzo-γ-pyrone structure, such as flavonols, flavones, and flavanones, and play a wide variety of biological roles in plants and other organisms (Kumar and Pandey, 2013; Hostetler et al., 2017). Flavone synthase (FNSI) catalyzes the conversion of flavanones, such as naringenin and eriodictyol to flavones, such as apigenin and luteolin (Falcone Ferreyra et al., 2015). Flavonoid metabolites produced by plants have varying effects on associated microbes. In *Arabidopsis*, DOWNY MILDEW RESISTANT 6 gene (DMR6) is involved in susceptibility to *Pseudomonas syringae*, and *dmr6* mutants do not produce apigenin, have high levels of salicylic acid (SA), and are resistant to *P. syringae*. *Arabidopsisdmr6* mutants complemented with *maize* FNSI regained susceptibility to *P. syringae* (Falcone Ferreyra et al., 2015), meaning that there is a link between flavones, production of SA, and susceptibility to pathogens. Bioassays using apigenin in the media have shown a dose-dependent reduction in growth of *Colletotrichum graminicola*, which suggested a phytochemical defense against fungi using flavones linked to the increase in production of naringenin and apigenin in both leaves and roots of maize infected with *C. graminicola* (Balmer et al., 2013).

Flavonoids and aflatoxin production have been studied by using *Aspergillus parasiticus* to inoculate kernels treated with a flavonoid mixture (0.39-mM naringenin, 0.24-mM neohesperidin, and 0.4-mM quercetin), which showed an 85–100% decrease in aflatoxin accumulation (Pok et al., 2020). Similar studies done with *A. flavus* incubated with anthocyanidins and related flavonoids showed inhibition of aflatoxin B1 production (Norton, 1999) and inhibition of growth (Li et al., 2019). TEM images of *A. parasiticus* treated with each flavonoid from the mix showed fungal damage such as, naringenin treatment-stimulated formation of lipid vesicles; neohesperidin treatment led to degradation or rupture of plasmalemma cell wall deformation and vesicle formation; quercetin treatment; agglutination of fibrillar layer, formation of dense grains in the inner wall, disruption of nuclear membranes, and formation of vesicles (Pok et al., 2020). Combination of the flavonoid mix led to greater degradation of membranes, organelles, and cytoplasm content (Pok et al., 2020). A study wherein *Fusarium culmorum and F. graminearum* were grown *in vitro* with naringenin, apigenin, luteolin, kaempferol, and quercetin showed that these fungi were capable of metabolizing these flavonoids to their derivatives, meaning that the fungi responded to the phytochemical defense from the plant (Bilska et al., 2018). In the same study, mycotoxin accumulation was significantly lowered if the fungi were treated with luteolin, kaempferol, naringenin, and apigenin, and, in this event, reduction could be controlled at the transcriptional level (Bilska et al., 2018).

Interestingly, there is a cross-talk between polyamines (PAs) and carotenoids/flavonoids in plants. Higher content of cellular spermidine (Spd) and spermine (Spm) in plants has strongly been correlated with increased biosynthesis of antioxidative compounds, e.g., carotenoids (Uarrota et al., 2018). Increased PA (Spd and Spm) content in tomato (*Solanum lycopersicum* L.) fruits was achieved by expressing a yeast (*Saccharomyces cerevisiae*) *S*-adenosylmethionine decarboxylase (*ySAMdc*) gene under a fruit-specific promoter (*E8*), which elevated lycopene (carotenoid) content in the transgenic fruits up to 2-fold (Mehta et al., 2002). The transgenic fruits with higher Spd and Spm content showed increased expression of flavonoid biosynthetic genes and increased cellular flavonoid content (Srivastava et al., 2007). Similar results were observed in transgenic tomato plants, expressing an apple [*Malus sylvestris* (L.) Mill. var. *domestica* (Borkh.) Mansf.] *Spds* gene under a constitutive promoter (*35S*). Transgenic fruits showed significantly increased (up to 2.2-fold) lycopene content accompanied by increased expression of several lycopene biosynthetic genes (Neily et al., 2011).

**FIGURE 5:**
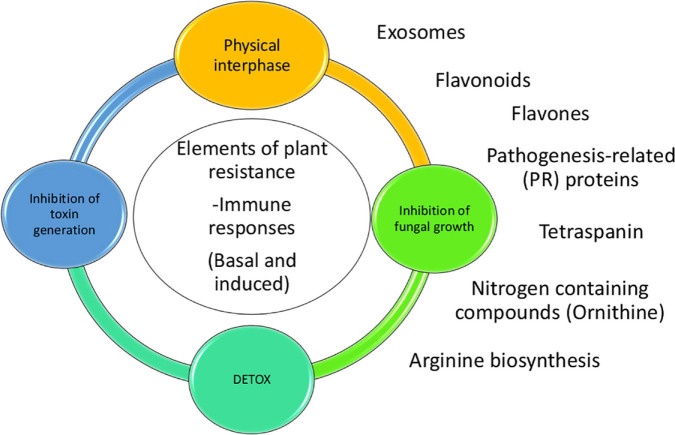
Model of the proposed plant responses to aflatoxin contamination. There are several elements of resistance to aflatoxin contamination such as events taking place in the physical interphase between fungi and kernel, inhibition of fungal growth, inhibition of toxin generation and detoxification. Some of the pathways and proteins tare are linked to the resistance to contamination are production of vesicles/exosomes, pathogenesis related proteins, tetraspanins, N-containing compounds (Ornithine), arginine biosynthesis and flavones/flavonoids. Flavonoids and flavones could be working in-between the physical interphase and the inhibition of fungal growth.

To conclude, we combined an analysis of transcriptomic data from aflatoxin-contamination-resistant and -susceptible maize lines with a genome-wide association analysis to examine the molecular mechanisms of maize resistance to *A. flavus* growth and aflatoxin contamination. Our study revealed activation of several important maize biochemical pathways and processes during *A. flavus* infection, including arginine biosynthesis, extracellular vesicle production, and flavonoid biosynthesis (Figure 5). It has been reported that polyamines, which arise from arginine biosynthesis, play an important role in *A. flavus* infection of maize (Uarrota et al., 2018). We confirmed that arginine is significantly correlated with defenses against aflatoxin contamination in kernels potentially due to its role as a precursor of polyamines. Future comprehensive omics studies of apoplastic fluid and extracellular vesicles isolated from resistant and susceptible maize lines will provide insight into their role in compartmentalization of defense-related compounds, such as polyamines and flavonoids, and how they function in response to fungal infection. Another area for further scientific exploration is the mode of action of several flavonoids produced by TZAR102 and their effect on fungal phenology and mycotoxin production. Evaluating the dynamics of flavonoid production in multiple maize [R] and [S] lines during *A. flavus* infection may lead to an effective flavonoid-based aflatoxin mitigation strategy.

## Data Availability Statement

The datasets presented in this study can be found in online repositories. The names of the repository/repositories and accession number(s) can be found below: The raw reads were submitted to NCBI and can be accessed under BioProject PRJNA767817.

## Author Contributions

LC-D, MG, ML, CC-W, and CS processed the samples. LC-D, MG, BM, ML, JC, and KR contributed to the data analysis. LC-D wrote the manuscript. All authors contributed to final draft, experimental design, and generation of figures.

## Conflict of Interest

The authors declare that the research was conducted in the absence of any commercial or financial relationships that could be construed as a potential conflict of interest.

## Publisher’s Note

All claims expressed in this article are solely those of the authors and do not necessarily represent those of their affiliated organizations, or those of the publisher, the editors and the reviewers. Any product that may be evaluated in this article, or claim that may be made by its manufacturer, is not guaranteed or endorsed by the publisher.
