# Supplementary material for: Flavonoids Modulate the Accumulation of Toxins From Aspergillus flavus in Maize Kernels
Source: Front Plant Sci. 2021 Nov 26;12:761446. doi: 10.3389/fpls.2021.761446 (PMC8662736; doi:10.3389/fpls.2021.761446)
Supplement: Supplementary file 4 [file Presentation_1.PPTX]

## Slide 1
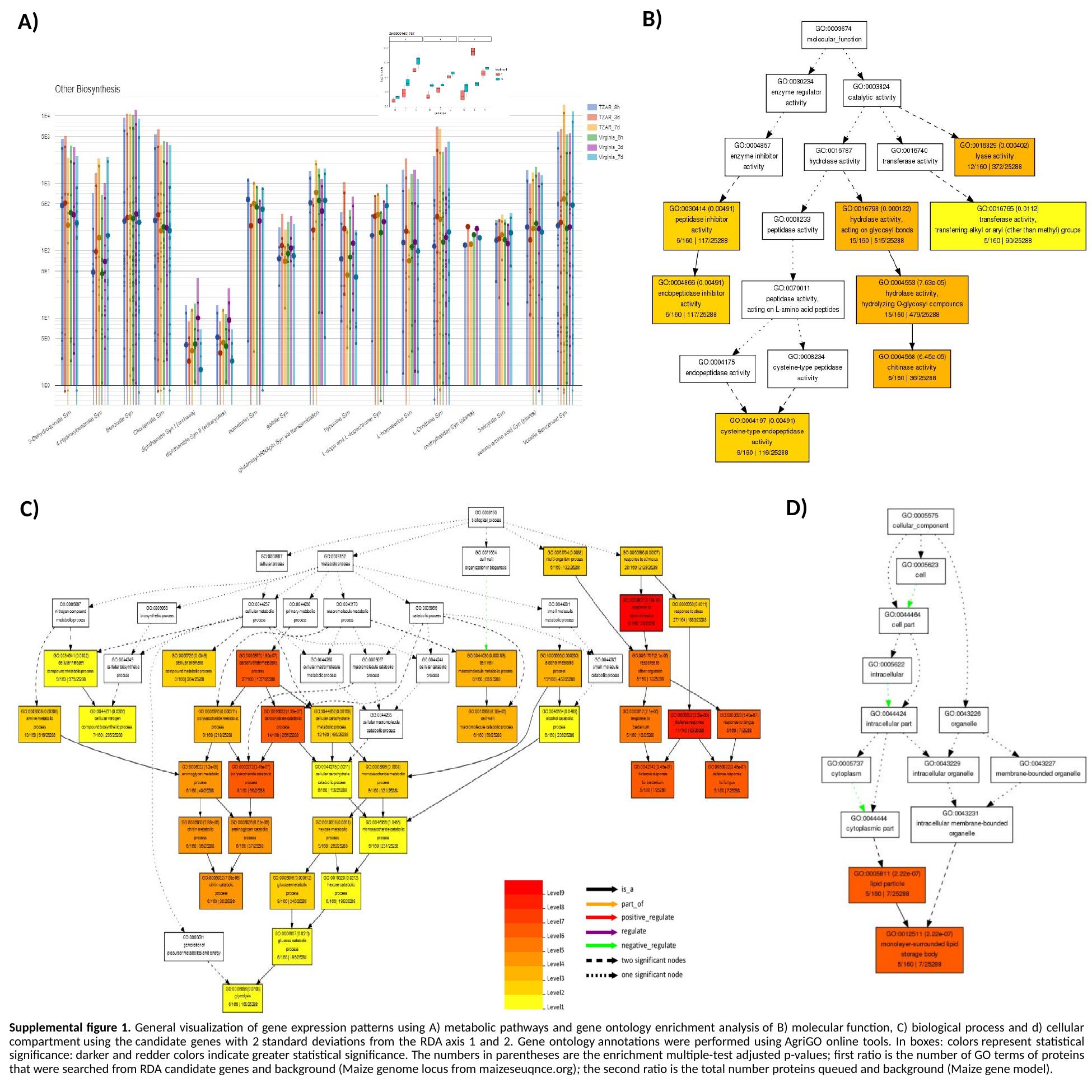

A)
B)
D)
C)
Supplemental figure 1. General visualization of gene expression patterns using A) metabolic pathways and gene ontology enrichment analysis of B) molecular function, C) biological process and d) cellular compartment using the candidate genes with 2 standard deviations from the RDA axis 1 and 2. Gene ontology annotations were performed using AgriGO online tools. In boxes: colors represent statistical significance: darker and redder colors indicate greater statistical significance. The numbers in parentheses are the enrichment multiple-test adjusted p-values; first ratio is the number of GO terms of proteins that were searched from RDA candidate genes and background (Maize genome locus from maizeseuqnce.org); the second ratio is the total number proteins queued and background (Maize gene model).

## Slide 2
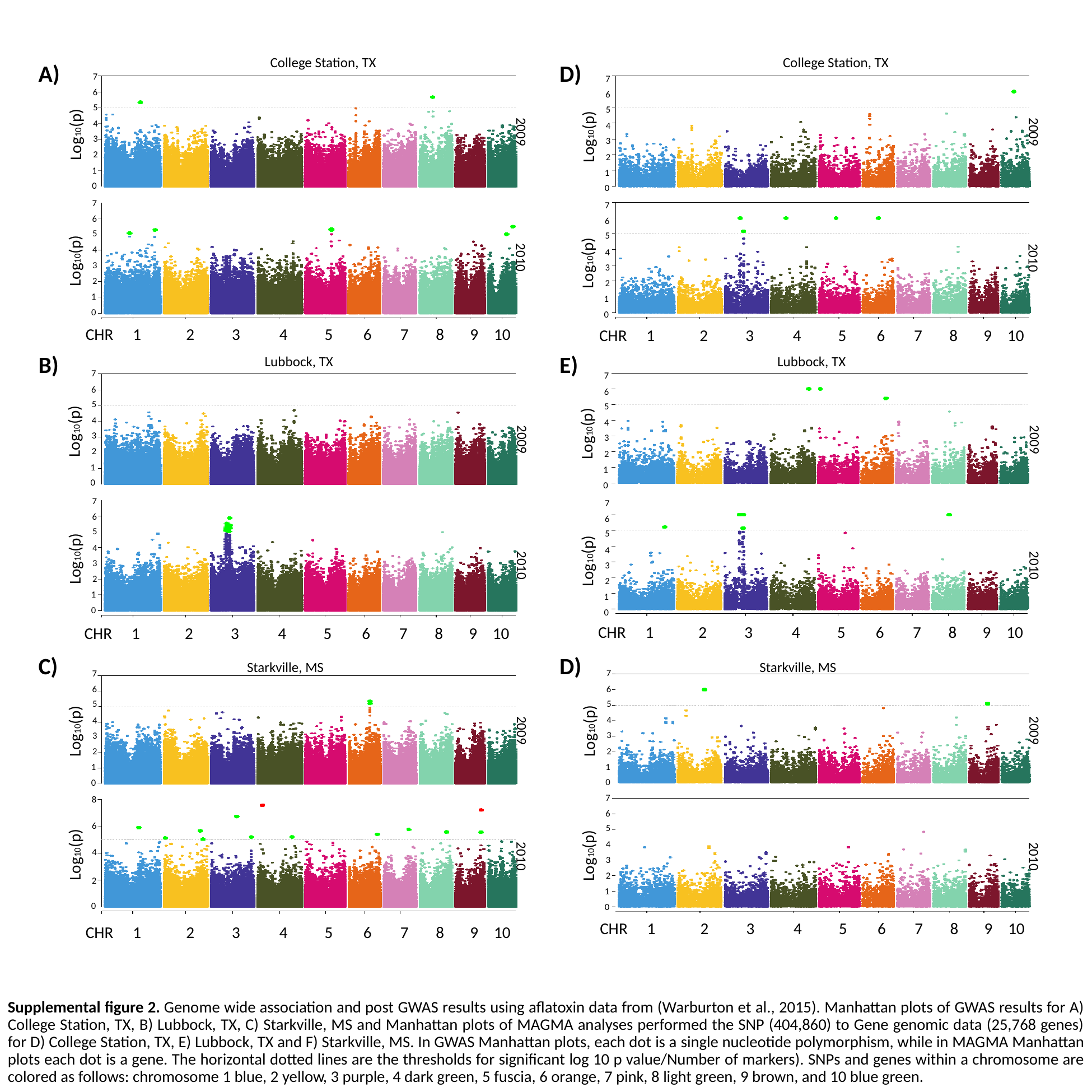

College Station, TX
College Station, TX
A)
D)
B)
E)
C)
D)
2009
2009
2010
2010
Lubbock, TX
Lubbock, TX
2009
2009
2010
2010
Starkville, MS
Starkville, MS
2009
2009
2010
2010
7
6
5
4
Log10(p)
3
2
1
0
7
6
5
4
Log10(p)
3
2
1
0
7
6
5
4
Log10(p)
3
2
1
0
7
6
5
4
Log10(p)
3
2
1
0
CHR
1
2
3
4
5
6
7
8
9
10
CHR
1
2
3
4
5
6
7
8
9
10
7
6
5
4
Log10(p)
3
2
1
0
7
6
5
4
Log10(p)
3
2
1
0
7
6
5
4
Log10(p)
3
2
1
0
7
6
5
4
Log10(p)
3
2
1
0
CHR
1
2
3
4
5
6
7
8
9
10
CHR
1
2
3
4
5
6
7
8
9
10
7
6
5
4
Log10(p)
3
2
1
0
7
6
5
4
Log10(p)
3
2
1
0
7
6
5
4
Log10(p)
3
2
1
0
8
6
4
Log10(p)
2
0
CHR
1
2
3
4
5
6
7
8
9
10
CHR
1
2
3
4
5
6
7
8
9
10
Supplemental figure 2. Genome wide association and post GWAS results using aflatoxin data from (Warburton et al., 2015). Manhattan plots of GWAS results for A) College Station, TX, B) Lubbock, TX, C) Starkville, MS and Manhattan plots of MAGMA analyses performed the SNP (404,860) to Gene genomic data (25,768 genes) for D) College Station, TX, E) Lubbock, TX and F) Starkville, MS. In GWAS Manhattan plots, each dot is a single nucleotide polymorphism, while in MAGMA Manhattan plots each dot is a gene. The horizontal dotted lines are the thresholds for significant log 10 p value/Number of markers). SNPs and genes within a chromosome are colored as follows: chromosome 1 blue, 2 yellow, 3 purple, 4 dark green, 5 fuscia, 6 orange, 7 pink, 8 light green, 9 brown, and 10 blue green.

## Slide 3
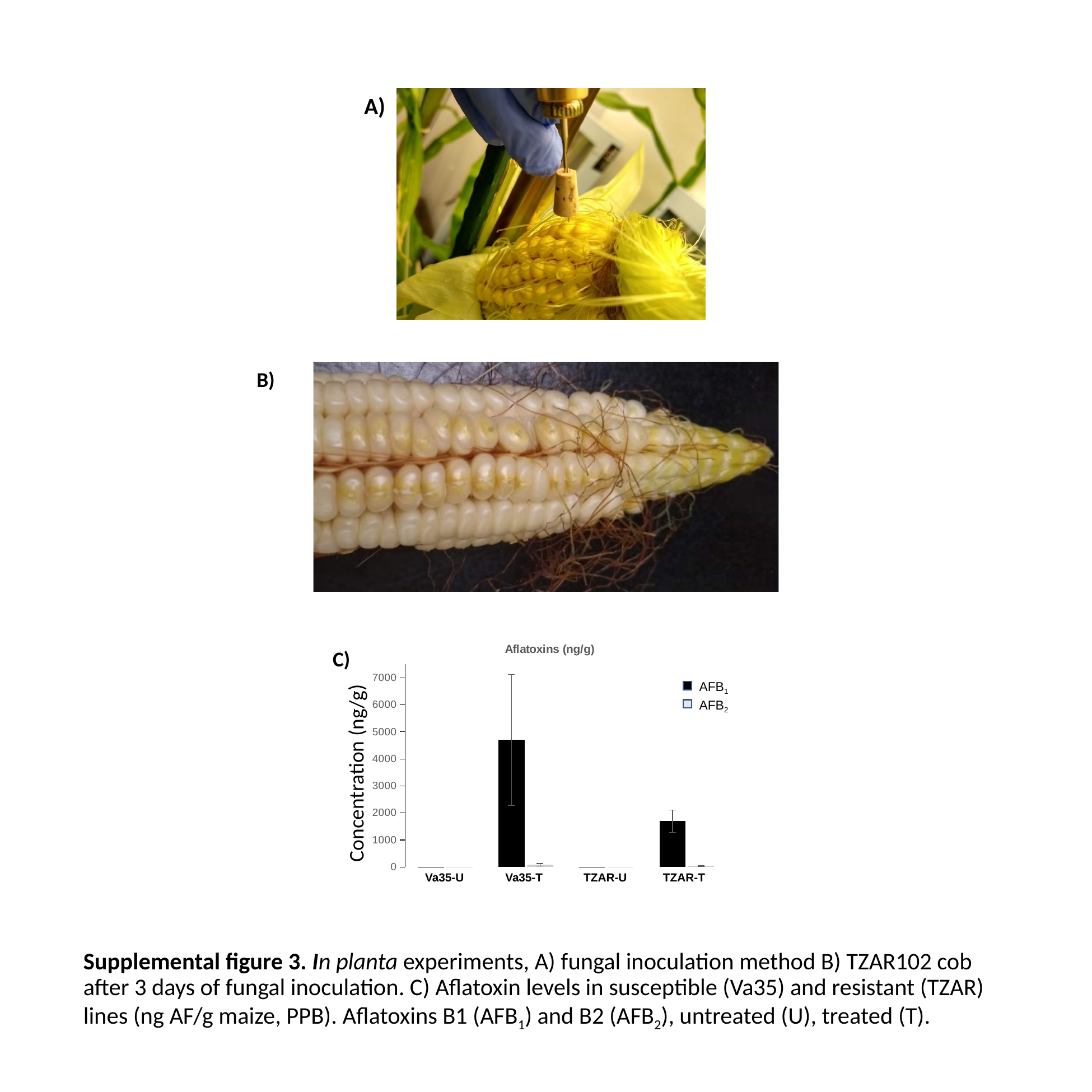

A)
B)
### Chart: Aflatoxins (ng/g)
| Category | AFB1 avg
SD | AFB2 avg
SD |
|---|---|---|AFB1
AFB2
Va35-U
Va35-T
TZAR-U
TZAR-T
C)
Concentration (ng/g)
Supplemental figure 3. In planta experiments, A) fungal inoculation method B) TZAR102 cob after 3 days of fungal inoculation. C) Aflatoxin levels in susceptible (Va35) and resistant (TZAR) lines (ng AF/g maize, PPB). Aflatoxins B1 (AFB1) and B2 (AFB2), untreated (U), treated (T).

## Slide 4
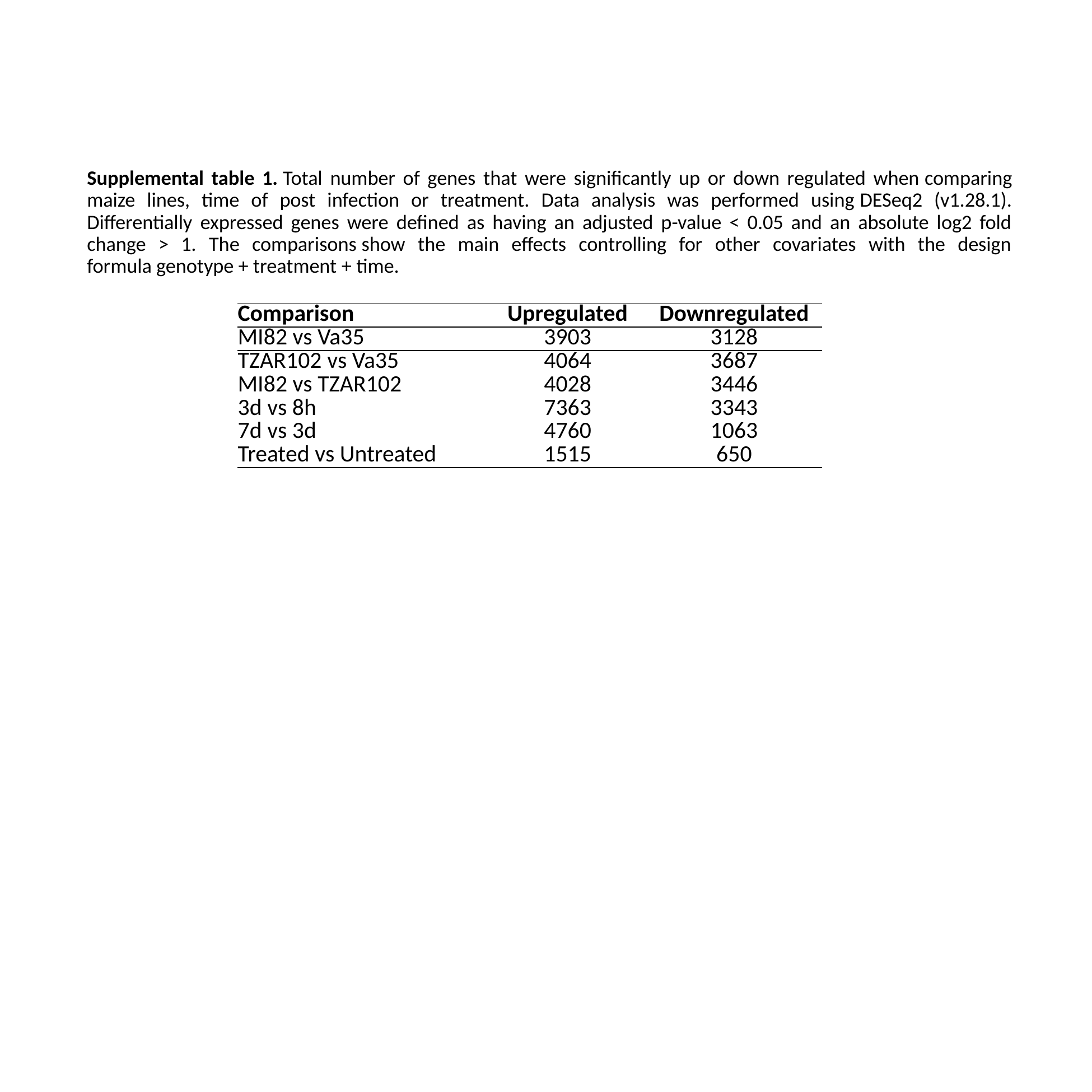

Supplemental table 1. Total number of genes that were significantly up or down regulated when comparing maize lines, time of post infection or treatment. Data analysis was performed using DESeq2 (v1.28.1). Differentially expressed genes were defined as having an adjusted p-value < 0.05 and an absolute log2 fold change > 1. The comparisons show the main effects controlling for other covariates with the design formula genotype + treatment + time.
| Comparison | Upregulated | Downregulated |
| --- | --- | --- |
| MI82 vs Va35 | 3903 | 3128 |
| TZAR102 vs Va35 | 4064 | 3687 |
| MI82 vs TZAR102 | 4028 | 3446 |
| 3d vs 8h | 7363 | 3343 |
| 7d vs 3d | 4760 | 1063 |
| Treated vs Untreated | 1515 | 650 |

## Slide 5
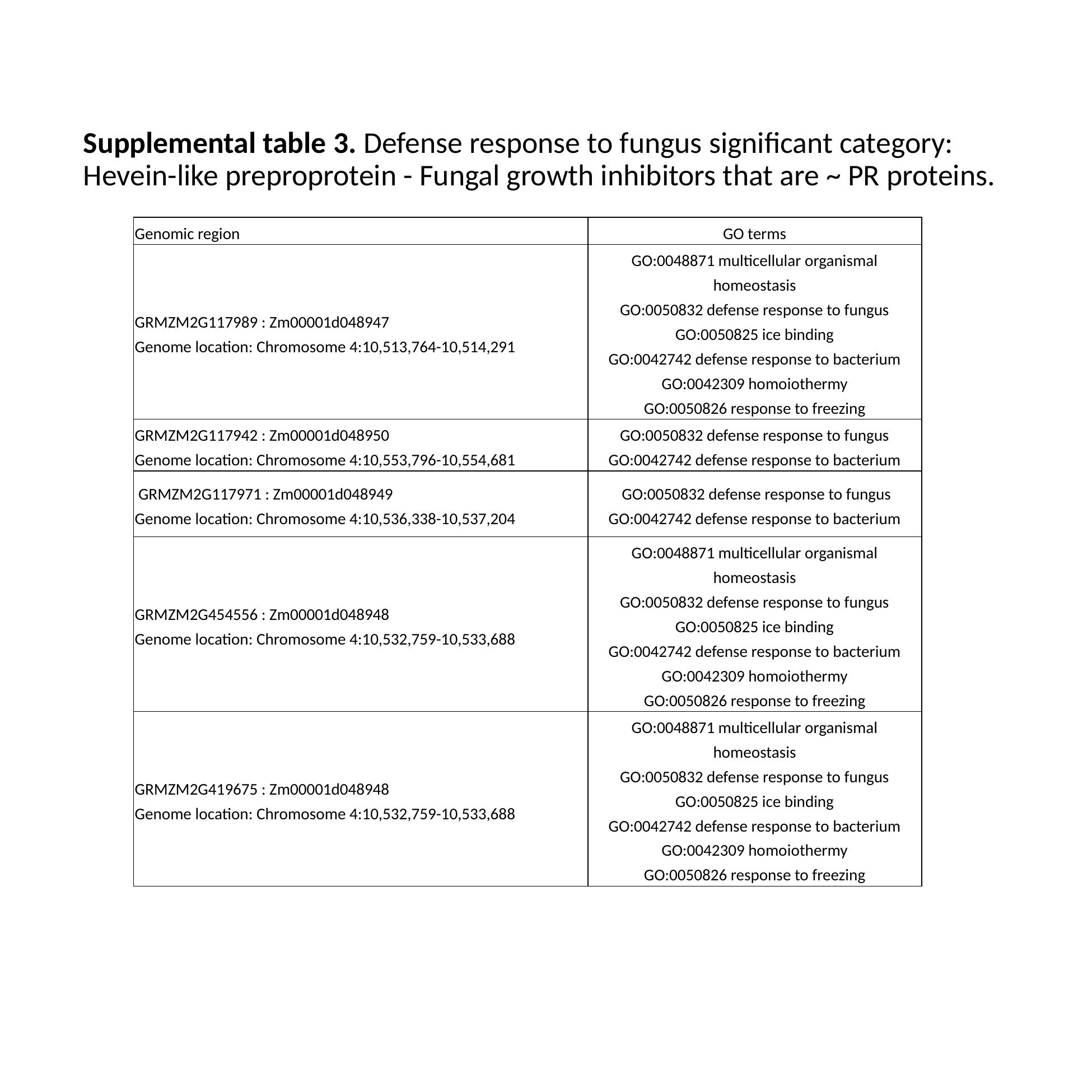

Supplemental table 3. Defense response to fungus significant category: Hevein-like preproprotein - Fungal growth inhibitors that are ~ PR proteins.
| Genomic region | GO terms |
| --- | --- |
| GRMZM2G117989 : Zm00001d048947 Genome location: Chromosome 4:10,513,764-10,514,291 | GO:0048871 multicellular organismal homeostasis GO:0050832 defense response to fungus GO:0050825 ice binding GO:0042742 defense response to bacterium GO:0042309 homoiothermy GO:0050826 response to freezing |
| GRMZM2G117942 : Zm00001d048950 Genome location: Chromosome 4:10,553,796-10,554,681 | GO:0050832 defense response to fungus GO:0042742 defense response to bacterium |
| GRMZM2G117971 : Zm00001d048949 Genome location: Chromosome 4:10,536,338-10,537,204 | GO:0050832 defense response to fungus GO:0042742 defense response to bacterium |
| GRMZM2G454556 : Zm00001d048948 Genome location: Chromosome 4:10,532,759-10,533,688 | GO:0048871 multicellular organismal homeostasis GO:0050832 defense response to fungus GO:0050825 ice binding GO:0042742 defense response to bacterium GO:0042309 homoiothermy GO:0050826 response to freezing |
| GRMZM2G419675 : Zm00001d048948 Genome location: Chromosome 4:10,532,759-10,533,688 | GO:0048871 multicellular organismal homeostasis GO:0050832 defense response to fungus GO:0050825 ice binding GO:0042742 defense response to bacterium GO:0042309 homoiothermy GO:0050826 response to freezing |
